# Effect of Increasing C/N Ratio on Performance and Microbial Community Structure in a Membrane Bioreactor with a High Ammonia Load

**DOI:** 10.3390/ijerph18158070

**Published:** 2021-07-30

**Authors:** Huaihao Xu, Yuepeng Deng, Xiuying Li, Yuxian Liu, Shuangqiu Huang, Yunhua Yang, Zhu Wang, Chun Hu

**Affiliations:** 1 Institute of Environmental Research at Greater Bay, Guangzhou Key Laboratory for Clean Energy and Materials, Key Laboratory for Water Quality and Conservation of the Pearl River Delta, Ministry of Education, School of Environmental Science and Engineering, Guangzhou University, Guangzhou 510006, China; xvhuaihao@163.com (H.X.); 2112004033@e.gzhu.edu.cn (Y.D.); 2112004050@e.gzhu.edu.cn (X.L.); 2111904034@e.gzhu.edu.cn (S.H.); yangyunhua@gzhu.edu.cn (Y.Y.); huchun@gzhu.edu.cn (C.H.); 2 Linköping University-Guangzhou University Research Center on Urban Sustainable Development, School of Environmental Science and Engineering, Guangzhou University, Guangzhou 510006, China; 3 State Key Laboratory of Pollution Control and Resource Reuse, School of the Environment, Nanjing University, Nanjing 210023, China

**Keywords:** nitrification, C/N ratio, membrane bioreactor, membrane fouling, microbial community

## Abstract

Herein, the responses of the operational performance of a membrane bioreactor (MBR) with a high ammonium-nitrogen (NH_4_^+^-N) load and microbial community structure to increasing carbon to nitrogen (C/N) ratios were studied. Variation in the influent C/N ratio did not affect the removal efficiencies of chemical oxygen demand (COD) and NH_4_^+^-N but gradually abated the ammonia oxidization activity of sludge. The concentration of the sludge in the reactor at the end of the process increased four-fold compared with that of the seed sludge, ensuring the stable removal of NH_4_^+^-N. The increasing influent COD concentration resulted in an elevated production of humic acids in soluble microbial product (SMP) and accelerated the rate of membrane fouling. High-throughput sequencing analysis showed that the C/N ratio had selective effects on the microbial community structure. In the genus level, *Methyloversatilis*, *Subsaxibacter*, and *Pseudomonas* were enriched during the operation. However, the relative abundance of ammonia-oxidizing bacteria (AOB) and nitrite-oxidizing bacteria (NOB) involved in nitrification declined gradually and were decreased by 86.54 and 90.17%, respectively, with influent COD increasing from 0 to 2000 mg/L. The present study offers a more in-depth insight into the control strategy of the C/N ratio in the operation of an MBR with a high NH_4_^+^-N load.

## 1. Introduction

With the acceleration of urbanization and agricultural specialization, high ammonia-nitrogen (NH_4_^+^-N) wastewater from cooking, food, aquaculture, and landfill leachate has led to increasingly acute nitrogen pollution in the water body [1]. In municipal solid waste treatment and some industrial production processes, such as the tanning of animal hides as well as petrochemical, pharmaceutical, and synthetic ammonia production, the wastewater produced may contain greater than 1000 mg/L NH_4_^+^-N, which is believed to be one of the primary pollutants causing the depletion of dissolved oxygen (DO) and eutrophication in water [2]. The increase in the content of inorganic nitrogen-containing compounds in ground and surface water around the world has given rise to the deterioration of water quality. Unfortunately, these pollutants have a significant impact on aquatic organisms and ultimately result in the contamination of freshwater, estuarine, and coastal marine ecosystems [3]. Therefore, nitrogen must be removed from wastewater before it is directly discharged into the environment, given its adverse effects [4].

Traditionally, nitrogen removal from wastewater is accomplished by two-step biological processes, i.e., nitrification and denitrification [5]. In this system, ammonia is oxidized to nitrate by nitrifying bacteria under aerobic conditions and then reduced to nitrogen by denitrifying bacteria under anoxic conditions [6]. These two steps are achieved by two types of autotrophic microorganisms called ammonia-oxidizing bacteria (AOB) and nitrite-oxidizing bacteria (NOB) [7]. As the first step, the oxidation of ammonia is deemed to be the rate-limiting step of the entire nitrification process; therefore, the final product of a typical nitrification process is usually nitrate [8].

Recently, many efforts have been made for the nitrification process of wastewater with NH_4_^+^-N levels greater than 1000 mg/L [9,10,11], whereas the biological treatment of high-strength ammonium wastewater remains challenging. Nitrifying microorganisms, including AOB and NOB, are autotrophs with slow growth and long doubling times [12,13]. Compared with heterotrophic bacteria, AOB and NOB are more sensitive to environmental factors, such as pH, temperature, and inhibitors [14]. A large amount of free ammonia (FA) produced by the high level of ammonium has a potent inhibitory effect on nitrifying bacteria [15,16,17]. Chai et al. [2] used an air-lift reactor to study the partial nitrification under high ammonium stress. It was found that the enhanced aeration due to the rise of influent NH_4_^+^-N led to an aggravation of sludge loss, thus stopping the effective operation. This indicated that if the sludge loss can be solved, the resistance of a reactor to high influent load can be improved.

Membrane bioreactor (MBR) technology, which combines the activated sludge method with membrane filtration technology, can retain almost all the suspended solids and biomass in the bioreactor using microfiltration (MF) or ultrafiltration membrane (UF) (pore size of 0.05–0.4 μm) for solid-liquid separation [18,19]. Therefore, the growth and enrichment of autotrophic nitrifying bacteria can benefit from the longer sludge retention time (SRT), the increased mixed liquid suspended solids (MLSS) concentration, and the subsequent reduced food-to-microorganisms (F/M) ratio in MBR [20].

The chemical oxygen demand (COD) that will further promote the growth and reproduction of heterotrophs that competes for DO with AOB and NOB is inevitable in ammonium-rich wastewater [21,22], hence reducing the proportion of AOB and NOB in the microbial community [23]. The influent C/N (COD/NH_4_^+^-N) ratio, one of the significant parameters in wastewater treatment, has been widely explored with regard to its impact on the biological nitrification process, but the levels of NH_4_^+^-N in previous studies were generally low [23,24,25,26]. Reports on the effect of different influent C/N ratios on the performance of the MBR with high-strength ammonium influent are limited. For example, Di Trapani et al. [27] used MBR and inoculated sludge from a municipal sewage treatment plant to treat the wastewater with an influent NH_4_^+^-N of 800 mg/L and a C/N ratio of 2.5. In this study, the removal rate of COD in the MBR was greater than 90%, whereas NH_4_^+^-N was only approximately 30% with a fast membrane fouling rate. The wastewater featured by high NH_4_^+^-N and COD content was directly treated without domestication, leading to the accumulation of NH_4_^+^-N in the reactor and the inhibition of FA on the activities of AOB and NOB [15,28,29], thereby affecting the nitrification process of the MBR. Hence, in a reactor in which the microbes are acclimated with heavy NH_4_^+^-N stress, it is of more practical significance to investigate the responses of the reactor performance to a variable influent C/N ratio under NH_4_^+^-N-enriched conditions.

An aerobic biological system is a complex microbial community ecosystem, in which AOB is only a class of specific functional microorganisms that contribute to the ammonia oxidation process, and many other microorganisms play an essential role in the removal of various pollutants, such as COD. Diverse microbial community structures in the sludge will be formed under different environmental conditions given that the microorganisms are easily affected by ecological factors [30,31]. The study of the microbial community structure of sludge is essential to reflect on the running state of the biological treatment system, explain the causes of some characteristic phenomena, and then supply data support for the optimization of the system operation. The recent rapid development of high-throughput sequencing technology provides a better choice for the investigation of the microbial community structure of samples in the complex environment given its advantages in sequencing depth and cost [32,33,34].

In some industries, NH_4_^+^-N and COD come from different pollution production units [35,36]; therefore, the C/N ratio entering a reactor can be regulated by influent flow. In this study, an MBR was operated with a high ammonia load rate (ALR), and the responses of reactor performance, microbial community structure, and the abundance of functional microbes to separate influent C/N ratios chosen according to the gradient in a certain range were assessed. This study addressed the following points: (i) whether the removal of conventional pollutants in MBR with a high ALR is affected by the increase in the influent C/N ratio, (ii) the development of membrane fouling with an increasing influent C/N ratio, and (iii) the effects of C/N ratios on the composition of microbial community structure and the abundance of functional microorganisms. Thus, this study offers more in-depth insight into the control strategy of the C/N ratio in the operation of MBR with a high NH_4_^+^-N load.

## 2. Materials and Methods

### 2.1. Experimental Set-Up

A submerged MBR was established with a working volume of 10 L and a hollow fiber membrane module that was made of polyvinylidene fluoride (PVDF) with a nominal pore size of 0.03 μm and an effective filtration area of 0.235 m^2^ was used in this research. The outlet of the membrane module was connected through the pump pipe with the suction pump that maintained the constant flow effluent, and a pressure sensor was installed between them to record the change of the transmembrane pressure in real-time during operation. The seed sludge came from an MBR that had run continuously and stably for a long time with an influent NH_4_^+^-N of 1000 mg/L and COD of 360 mg/L in the laboratory. The artificial simulated wastewater prepared based on tap water contained NH_4_Cl (as 1000 mg/L NH_4_^+^-N) and KH_2_PO_4_ (as 3 mg/L TP) as basic nutrients and essential trace elements (5 mg/L MgSO_4_·7H_2_O, 2 mg/L Fe_2_(SO_4_)_3_, 0.5 mg/L H_3_BO_3_, 0.4 mg/L ZnCl_2_, 0.4 mg/L (NH_4_)_6_Mo_7_O_24_·4H_2_O, 0.4 mg/L NiCl_2_·6H_2_O, 0.5 mg/L AlCl_3_·6H_2_O, 0.3 mg/L CoCl_2_·6H_2_O, 0.4 mg/L CuSO_4_·5H_2_O, 0.4 mg/L MnCl_2_·4H_2_O). NaHCO_3_ (12,500 mg/L, m(NaHCO_3_):m(NH_4_^+^-N) = 12.5) was formulated to provide the alkalinity required for the nitrification process, thus maintaining the pH of the reaction mixture within a reasonable range (7–8). The variation of influent COD concentration was realized by changing the amount of glucose added. All the reagents were of analytical purity. A blast aeration system was applied to maintain the DO at 2–6 mg/L in the reactor at room temperature (20 ± 5 °C). The experimental period during which the hydraulic retention time (HRT) was set to 14 h, and no sludge was drawn from the MBR for a 100-day period, which was divided into four stages. The operating conditions of the reactor at each stage are presented in Table 1. When the transmembrane pressure reached 30 kPa, the membrane module was removed from the MBR for physical and chemical cleaning [37].

### 2.2. Batch Experiments

Batch experiments were conducted to investigate the ammonia oxidization rate (AOR) of seed sludge at different initial COD conditions and the sludge cultured in the reactor at each stage, separately. A reaction was performed at 25 °C in a conical flux with a total volume of 200 mL and a calculated amount of sludge to be tested to ensure that the final MLSS was 2000 mg/L. The initial concentration of NH_4_^+^-N was regulated by adding NH_4_Cl. NaHCO_3_ was provided to ensure the required alkalinity for the nitrification process, and other factors of a specific initial amount needed for the experiment were also provided at the same time. DO was controlled at 2–4 mg/L by an air compressor through porous diffuser stones, and the solid-liquid mixture was maintained as a uniform mixture using a magnetic stirrer. Samples were collected periodically and centrifuged at 3000 rpm to measure NH_4_^+^-N in the supernatant. The AOR value of sludge, which expressed as mg NH_4_^+^-N/(g MLSS·h), can be calculated based on the curve of NH_4_^+^-N concentration versus time.

### 2.3. Analytical Methods

Determination of MLSS and mixed liquor volatile suspended solids (MLVSS) of sludge as well as the COD, NH_4_^+^-N, NO_2_^–^-N, and NO_3_^–^-N in the effluent were assessed using standard methods. The effluent parameters of MBR under different C/N ratios were analyzed by one-way ANOVA using Excel software. Significance level (*p* < 0.05) was accepted for the statistical tests. After the effluent of MBR was filtered using a glass fiber membrane with 0.45-micrometer pore size, the composition of soluble microbial products (SMP) was characterized using a three-dimensional (3D) excitation-emission matrix (EEM) spectra [38]. EEM spectra of SMP were obtained using a fluorescence spectrophotometer (F-7000, Hitachi High-Technology Corp., Chiyoda-ku, Tokyo, Japan) by setting the excitation wavelength range from 200 to 450 nm with 5-nanometer increments and the emission wavelength range from 280 to 550 nm with 1-nanometer increments. An emission cutoff filter for 290 nm was used to eliminate the interference of the second-order Rayleigh scattering to the fluorescence spectra.

### 2.4. DNA Extraction and PCR

DNA of sludge samples collected at different stages was extracted by using FastDNA SPIN Kit for Soil (MP Biomedicals, Irvine, CA, USA) according to the instructions. DNA concentration and purity were evaluated with gel electrophoresis and microspectrophotometry (Nano Drop, ND-2000, Waltham, MA, USA). The former primer 27F (5′-AGAGTTTGATYMTGGCTCAG-3′) and the reverse primer 338R (5′-TGCTGCCTCCCGTAGGAGT-3′) that target the hypervariable V1–V2 region of 16S rRNA gene were responsible for amplification of extracted DNA [39]. To sequence different samples in one channel, barcodes were inserted between the adapter and the forward primer. A polymerase chain reaction (PCR) system with a total volume of 50 µL included 25 µL of 2× EasyTaq^®^ PCR SuperMix (Transgene, Beijing, China), 2 µL of former and reverse primers (10 µM), respectively, 40 ng template DNA and ddH_2_O. DNA amplification was performed as follows: initial denaturation at 98 °C for 5 min; 20 cycles of denaturing at 98 °C for 30 s, annealing at 50 °C for 30 s, and elongation at 72 °C for 40 s; and a final extension at 72 °C for 10 min. The purity of PCR products was tested using gel electrophoresis after purification with the TaKaRa Mini BEST DNA Fragment Purification Kit Ver. 4.0 (Takara, Kusatsu, Shiga, Japan).

### 2.5. Sequencing and Analysis

High-throughput sequencing was performed at Zhongyijinda Analytical & Testing Co., Ltd. (Yixing, China) on Illumina Miseq platform. Mothur (http://www.mothur.org/) was applied to select the corresponding sequence with specific barcodes inside each sample. Data denoising was accomplished using bioinformatics software Mothur and Sickle (https://github.com/najoshi/sickle) following the method of a previous study [40]. To compare all the samples at the same sequencing depth, the number of valid sequences was unified by randomly drawing 28,716 reads from the samples after noise reduction. The Ribosomal Database Project (RDP) (http://rdp.cme.msu.edu/) Classifier was responsible for downstream taxonomic assignments with a confidence threshold of 50% [41]. Richness and diversity indices of microorganisms, including operational taxonomic units (OTUs), Chaos index, and Shannon index, were calculated using Mothur [42]. Software Paleontological Statistics (PAST, v.3.01) was used for cluster analysis of the microbial community with an unweighted pair-group average method.

## 3. Results and Discussion

### 3.1. Effect of C/N Ratio on Performance and Sludge Characteristics of MBR

**Figure 1:**
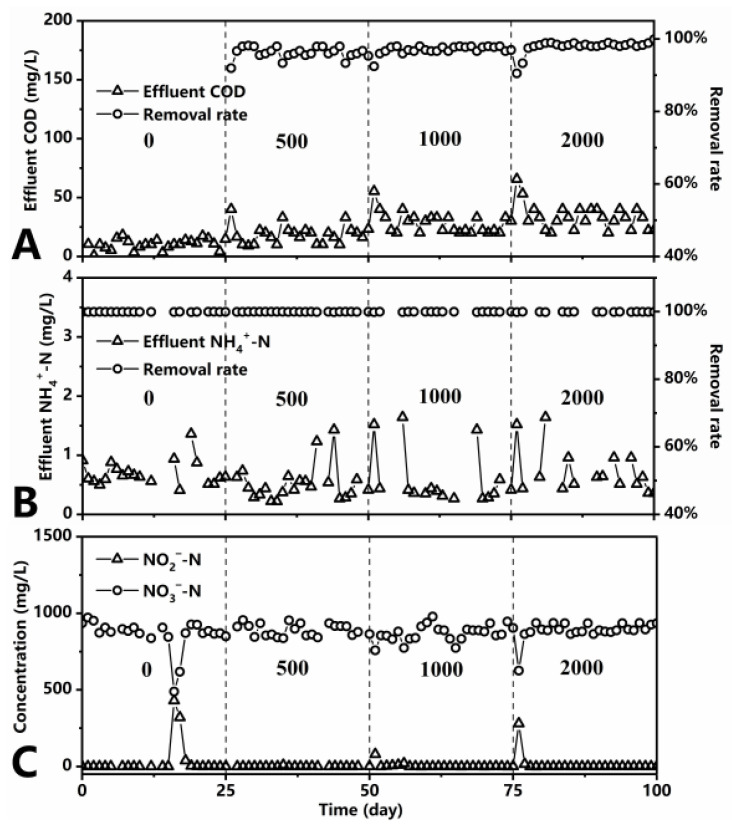
Effect of different C/N ratios on water quality parameters of effluent ((**A**): COD; (**B**): NH_4_^+^-N; (**C**): NO_2_^−^-N and NO_3_^−^-N).

The MBR operation was divided into four stages, each of which ran for 25 days continuously. The effects of 0, 500, 1000, and 2000 mg/L influent COD at each stage successively on the performance of MBR were investigated as influent NH_4_^+^-N was maintained at 1000 mg/L. As shown in Figure 1, the effluent COD content of the reactor was relatively low at each stage, although it increased accordingly (ANOVA, *p* < 0.05, Table 2), providing a satisfactory COD removal rate; this finding might be explained by the fact that the COD in the synthetic wastewater was glucose, which is a biodegradable substance. Greater than 99% of NH_4_^+^-N was removed, as the effluent concentration was less than 2 mg/L during the whole period (ANOVA, *p* > 0.05, Table 2), indicating that different influent COD contents did not affect NH_4_^+^-N removal. The effluent NO_2_^–^-N concentration was also generally less than 2 mg/L, with the exception of aeration device failures on the 16th and 76th day that led to insufficient DO in the reactor, resulting in a brief accumulation of NO_2_^−^-N (ANOVA, *p* > 0.05, Table 2). The reason for nitrite accumulation was that the Monod saturation constants of oxygen for AOB and NOB are 0.3 and 1.1 mg/L, respectively [43]. These findings indicate that AOB has a stronger affinity for DO, and NOB is initially inhibited when DO is insufficient [44,45,46]. The NO_3_^−^-N concentration in the effluent revealed that most of the NH_4_^+^-N in the influent was converted into NO_3_^−^-N (ANOVA, *p* > 0.05, Table 2). Thus, the four different C/N ratios had a negligible effect on the treatment efficiency of MBR, which demonstrated excellent performance for the removal of NH_4_^+^-N and COD with a high NH_4_^+^-N load. The study by Xia et al. [23] also showed that the removal rate of COD and NH_4_^+^-N in the reactor changed marginally when the C/N ratio increased from 3 to 5 and 10.

**Figure 2:**
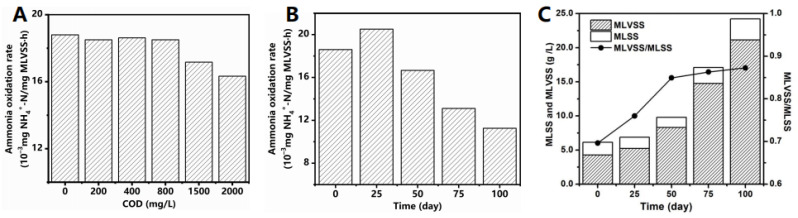
(**A**) Ammonia oxidation rate of seed sludge under different initial COD concentrations. (**B**) Ammonia oxidization rate of sludge at different stages. (**C**) Variation of the sludge concentration in the MBR.

The effects of different initial COD values of 0, 200, 400, 800, 1500, and 2000 mg/L on the AOR of seed sludge were investigated in batch experiments. Figure 2A demonstrates that when the initial COD content was less than 800 mg/L, it did not affect the AOR of the sludge, which reached 18.60 × 10^−3^ mg NH_4_^+^-N/(mg MLVSS·h). When 1500 and 2000 mg/L were attained, the initial COD concentrations had specific effects on the AOR of the seed sludge, which decreased by 7.71 and 10.60%, respectively, but remained at a high level. The degradation of COD was achieved by heterotrophs, whereas autotrophic AOB was mainly responsible for the oxidation of NH_4_^+^-N. The high COD content promoted the activity of heterotrophs and might cause the competitive inhibition of AOB, i.e., competition for DO [21,22], thus affecting the activity of AOB. In batch experiments, a high COD stress mainly affected the microbial activity. In contrast, the composition of the microbial community and the proportion of AOB would be dramatically changed in continuous flow experiments [23]. Consequently, the AOR of the sludge stabilized at each operation stage of the MBR was researched.

Figure 2B shows the variation of the AOR of the sludge in the reactor during the operation. Accordingly, when the influent COD content was 0 mg/L, the AOR increased from 18.60 × 10^−3^ mg NH_4_^+^-N/(mg MLVSS·h) of the seed sludge to 20.50 × 10^−3^ mg NH_4_^+^-N/(mg MLVSS·h). When the influent COD concentrations were 500, 1000, and 2000 mg/L, the AOR values of the sludge stabilized in MBR were 16.66, 13.11, and 11.26 × 10^−3^ mg NH_4_^+^-N/(mg MLVSS·h), which declined by 18.77, 36.08, and 45.09%, respectively. Thus, the AOR of the sludge stabilized at each stage diminished significantly as the influent COD content increased, which was consistent with the conclusions of Ballinger et al. [24]. The reduction in the AOR of the sludge in MBR exceeded that of the seed sludge in the batch experiments, which verified that a high COD stress had a greater effect on continuous flow experiments. Although the AOR of the sludge decreased by 45.09% when the influent COD content was 2000 mg/L, the removal rate of NH_4_^+^-N in MBR was maintained at greater than 99% during the whole period, which was due to the non-discharge of sludge, and the increase in the sludge concentration ensured the stable removal rate.

The variation in the sludge concentration in the MBR is presented in Figure 2C. The MLSS concentration in the reactor increased slowly from 6143 mg/L of seed sludge to 6870 mg/L at stage I. The increase in the influent COD content rapidly increased the sludge concentration, causing the MLSS and MLVSS concentrations to eventually reach 24,224 and 21,141 mg/L, respectively. Figure 2C also shows that the ratio of MLVSS/MLSS increased steadily during the operation period from 0.70 of seed sludge to 0.87 at the end, indicating an increase in the proportion of organic components in the sludge composition. The increase in the sludge concentration played a vital role in the stable removal of pollutants.

### 3.2. Membrane Fouling and SMP

**Figure 3:**
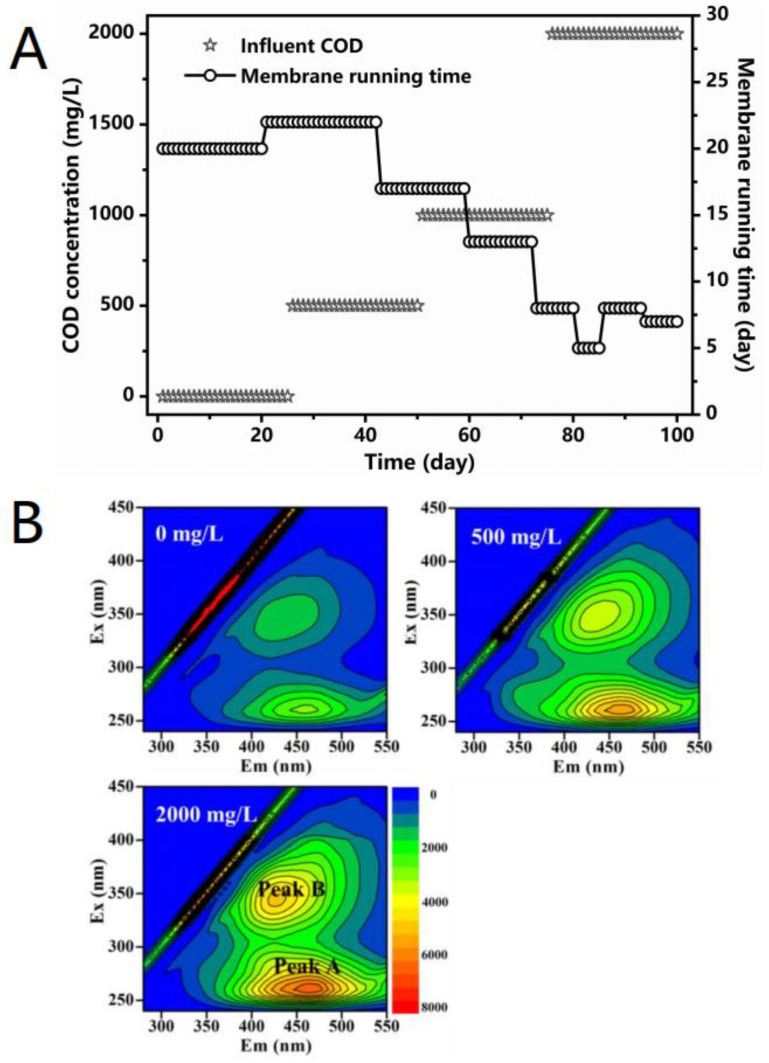
(**A**) Membrane fouling of the MBR during operation. (**B**) EEM spectra of SMP in the effluent of the MBR.

Membrane fouling refers to the phenomenon whereby sludge floc, dissolved organic matter, and colloidal particles in the mixture are deposited and adsorbed on the membrane through physical and chemical interactions [47,48]. These fouling processes lead to the reduction in membrane pore size and even blockage, representing a bottleneck restricting the development of MBR [49]. The membrane fouling observed during the operation was characterized by transmembrane pressure (TMP). The faster the TMP increased, the faster the membrane fouling rate was. It was necessary to replace the contaminated membrane module when the TMP exceeded 30 kPa, given that it was difficult to ensure a stable effluent for this membrane [37]. The duration of the membrane module during the MBR operation is presented in Figure 3A. The membrane module was used for more than 20 days in stages I and II. The service time of the membrane module decreased to 13 days because the influent COD concentration was increased to 1000 mg/L in stage III, and the service time further declined to 7 days in stage IV. These results demonstrate that when the influent NH_4_^+^-N concentration was kept constant, the increase in the C/N ratio accelerated the membrane fouling rate. On the contrary, Feng et al. [50] found that the membrane fouling rate was increased in the context of a low C/N ratio when the influent COD concentration was kept constant. In the case of a constant influent COD concentration, a lower C/N ratio meant a higher influent NH_4_^+^-N concentration, which may be the main factor to accelerate the membrane fouling.

SMP is a soluble extracellular polymeric substance (EPS), which generally refers to all the soluble organic compounds released by microbes in the normal process of metabolism or death, such as proteins, humic acids, and polysaccharides [51,52,53]. The peptides in SMP are naturally adsorbed by the membrane pores, resulting in membrane pore blockage and irreversible fouling; polysaccharides and proteins can complex with metal cations and adsorb to the membrane surface to form relatively dense and stable gel layer fouling [53]. SMP is an essential cause of membrane fouling in MBR [18,54,55,56], and the membrane flux decreases as the SMP concentration increases. Proteins cause more reduction in membrane flux than carbohydrates [55]. Humic acids have a lower molecular weight (*Mw*) than polysaccharides and proteins and contribute minimally to membrane fouling [49].

EEM is a fast and sensitive tool, which can provide fingerprint information of fluorescent substances and has been widely used in the characterization of SMP [38,57]. The EEM spectra of SMP in the effluent of MBR are shown in Figure 3B. The two main characteristic peaks of SMP are produced by activated sludge. Peak A (Ex/Em: 260/460–464) and Peak B (Ex/Em: 345–350/426–437) were both attributed to humic acids [38]. Table 3 shows that the intensity of these two characteristic peaks increased as the influent COD content increased. When the influent concentration of COD reached 2000 mg/L, the intensity of the two characteristic peaks was greater than double that of 0 mg/L. It could be concluded that high influent COD stress promoted the production of SMP, consequently accelerating the rate of membrane fouling.

### 3.3. Microbial Community Structure

To observe the effect of different C/N ratios on the microbial community structure of sludge, activated sludge was collected from the reactor at three time-points on the 25th, 75th, and 100th days of the MBR operation, which were labelled C0, C1000, and C2000, respectively. In addition, samples of the filter cake layer of the membrane module (marked as M) collected at the end of the operation of the reactor (on the 100th day) and the seed sludge were also analyzed to explore the microbial characteristics.

**Figure 4:**
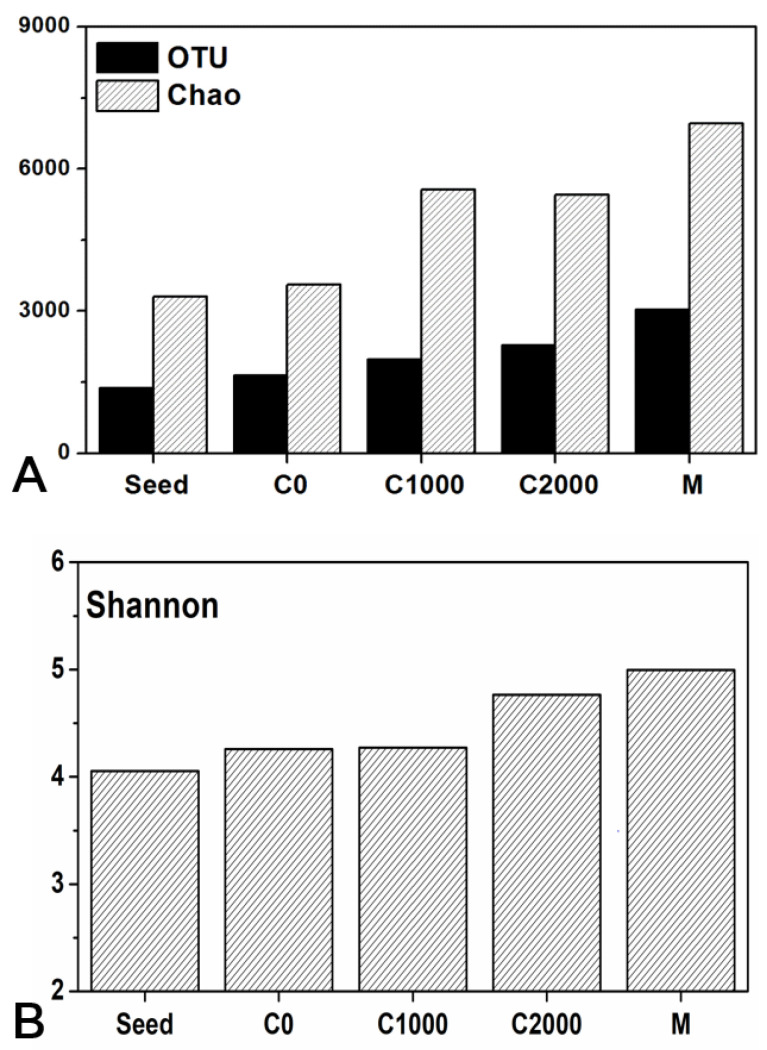
(**A**) Microbial richness and (**B**) diversity index of all sludge samples.

Figure 4 presents the richness and diversity index of each sample, demonstrating that the microbial richness and diversity index of the seed sludge was the lowest with OTU, Chaos, and Shannon values of 1394, 3312, and 4.03, respectively. The seed sludge came from the MBR, which had run stably for a long time under the same conditions, thus forming a stable and low diversity microbial community structure. As the operating conditions changed, the reduction in influent COD further promoted the reproduction of autotrophic microorganisms. Simultaneously, the heterotrophs were still able to survive using the metabolites produced by autotrophs [53]; therefore, the microbial diversity and richness of sample C0 increased. Since then, the increase in the influent COD content promoted the growth of heterotrophs, which further increased the richness and diversity of sludge microbes. Some of the microorganisms in the filter cake layer were intercepted by the membrane module, which could also provide attachment points for microorganisms to form biofilms. This particular habitat facilitated the increased richness and diversity of the microbes in the cake layer.

**Figure 5:**
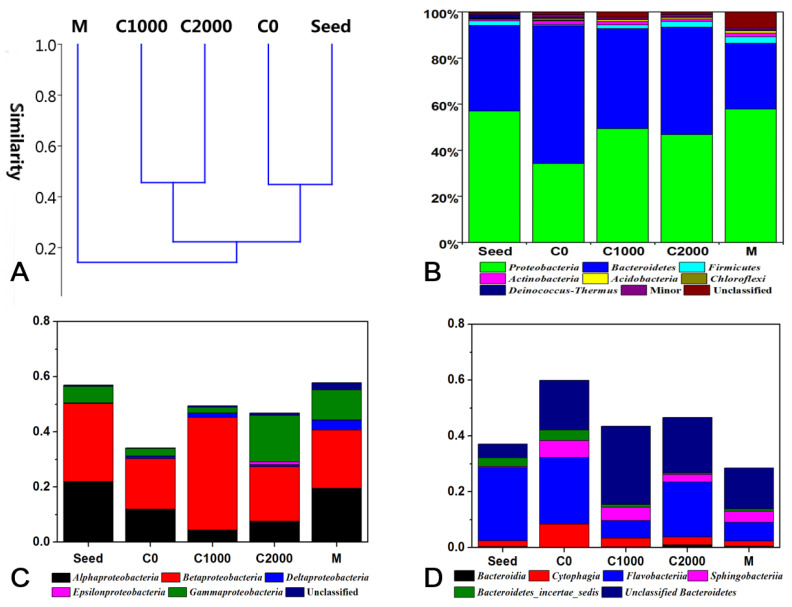
(**A**) Cluster analysis of microbial community structure. (**B**) Relative abundance of bacterial phyla. (**C**) Relative abundance of bacterial classes in *Proteobacteria*. (**D**) Relative abundance of bacterial classes in *Bacteroidetes*.

The differences in microbial communities in different samples were characterized using cluster analysis. Figure 5A demonstrates that the microbial community structure of the seed sludge was similar to that of sample C0. In addition, sample C1000 was similar to C2000, and that of the filter cake layer sample M was the most different from the other samples. The seed sludge came from a high NH_4_^+^-N loading reactor with a relatively low C/N ratio (0.36); therefore, it was similar to sample C0. Samples C1000 and C2000 were similar sludge samples with high C/N ratios. Ecological conditions, such as the DO of microbes in the cake layer, were quite different from those of the reactor mixture; thus, the community structure of sample M was separated from that of the other sludge samples.

The bacteria in the sludge and filter cake layer of membrane modules mainly belonged to phyla *Proteobacteria* and *Bacteroidetes*. The total abundance of these two bacterial phyla in the sludge sample and in the cake layer was greater than 90 and 85%, respectively (Figure 5B). *Proteobacteria* is the most abundant phylum of bacteria, and this phylum typically occupies a dominant position in sewage treatment plants and exhibits a very high abundance. Zhang et al. [41] investigated the microbial community structure of 14 sewage treatment plants and found that phylum *Proteobacteria* had the highest abundance in all the samples tested, accounting for 36–65%. Several pathogenic genera in *Proteobacteria* are involved in the nitrification process, including AOB *Nitrosomonas* and *Nitrosospira* belonging to class *Betaproteobacteria*, *Nitrosococcus* belonging to class *Gammaproteobacteria*, and NOB *Nitrobacter* [58,59]. *Bacteroidetes* is also a typical microorganism found in sewage treatment [41], which contributes to the removal of COD because its genome contains several functional genes related to the degradation of proteins and carbohydrates. The genome of *Bacteroidetes* is malleable and often reorganizes, which helps these bacteria adapt to different niches, thus, *Bacteroidetes* are dominant bacteria in soil, the ocean, and the intestinal tract [60].

The abundance of *Bacteroidetes* in sample C0 was the highest at 59.92%. A low COD load was beneficial to the enrichment of *Bacteroidetes*, whereas the abundance levels of phyla *Proteobacteria* and *Firmicutes* were in contrast to that of *Bacteroidetes*. Unclassified bacteria accounted for 6.97% of microbes in the filter cake layer, far exceeding the average proportion of 1.32% in the sludge samples. In this study, the investigation of the microbial community structure using a high-throughput sequencing technique was based on similarity comparisons with known microbial sequences. Previously, a large number of studies have assessed the microbial community composition of activated sludge, but few studies have focused on the microbial distribution in the cake layer of the membrane module, which may explain the difference in the unclassifiable proportion of the two types of samples. In addition, phyla *Actinobacteria* and *Acidobacteria* were also abundant in the samples.

Furthermore, the composition of dominant phyla was analyzed at the class level, and phylum *Proteobacteria* was mainly composed of the following three classes: *Alphaproteobacteria*, *Betaproteobacteria*, and *Gammaproteobacteria*. In addition, the abundance of classes *Deltaproteobacteria* and *Epsilonproteobacteria* were low, and class *Zetaproteobacteria* was not detected in all the samples (Figure 5C). Among them, class *Betaproteobacteria* predominated in all the samples, and class *Alphaproteobacteria* exhibited a higher abundance in the seed sludge and the filter cake layer compared with the other three samples. The abundance of class *Gammaproteobacteria* in the samples with high influent COD content was dramatically increased compared with the other samples, demonstrating that increasing the influent COD concentration promoted the enrichment of these microorganisms. Class *Deltaproteobacteria* exhibited an abundance of 3.51% in the cake layer, which was considerably increased compared with that in the other sludge samples with an average of 0.85%. This finding indicates that this type of microbe was easily intercepted by and attached to membrane modules. The proportion of *Proteobacteria* phylum that could not be classified in the cake layer was also higher. A total of five classes were detected in phylum *Bacteroidetes* (Figure 5D), among which class *Flavobacteriia* predominated in all the samples. The abundance of class *Cytophagia* in the sludge sample with an influent COD of 0 mg/L was significantly increased compared with the other samples. However, the class *Sphingobacteriia* had the lowest abundance in the seed sludge but higher abundance in the other samples, demonstrating that *Sphingobacteriia* was enriched in MBR. A high proportion of unclassified *Bacteroidetes* was noted in the sludge and cake layer samples, revealing that some *Bacteroidetes* that had not been adequately studied are enriched in the reactor.

**Figure 6:**
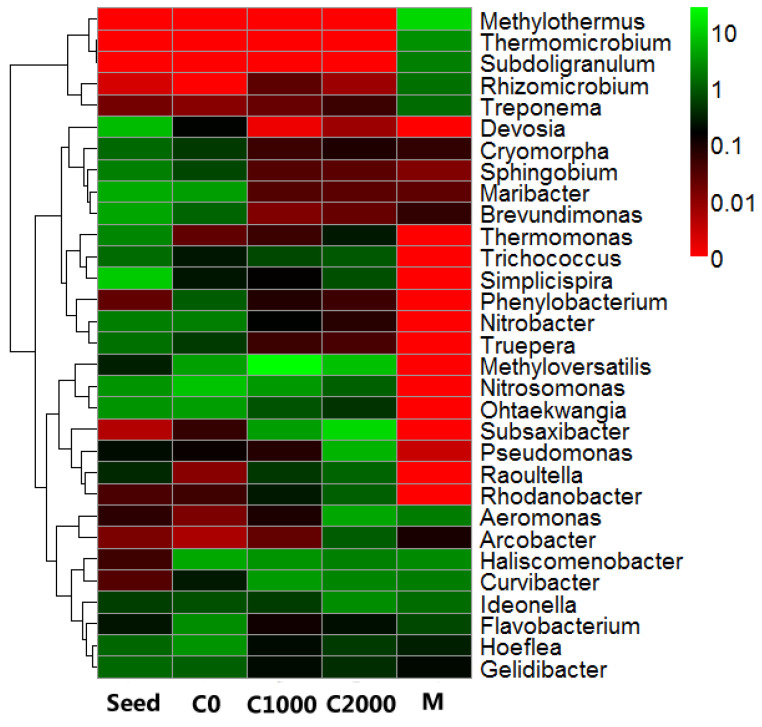
Heatmap of the relative abundance of bacterial genera in all samples.

The composition of microbes at the genus level is shown in Figure 6. Accordingly, genera *Methyloversatilis*, *Subsaxibacter*, *Aeromonas*, *Pseudomonas*, *Curvibacter*, and *Ideonella* were enriched in samples with a high C/N ratio. However, genera *Devosia*, *Brevundimonas*, *Ohtaekwangia*, *Simplicispira*, *Maribacter*, and *Thermomonas* that had relatively high abundances in the seed sludge were diminished visibly in the sludge samples with high C/N ratios. *Methyloversatilis* is a type of heterotrophic bacteria [61]; therefore, its growth benefited from a high C/N ratio, explaining its extremely high abundance. In addition, the microbial community structure of the filter cake layer of the membrane module was considerably different from that of all the sludge samples, including the corresponding C2000 sludge sample collected from the same day. In the cake layer, the genus *Methylothermus*, which was not detected in the other four sludge samples, had the highest abundance at 12.18%. Genera *Treponema*, *Rhizomicrobium*, *Thermomicrobium*, and *Subdoligranulum* also had relatively high abundances in the cake layer with low abundance in the sludge samples. Moreover, some of these genera were even not detected in the sludge samples. The results showed that these microbes easily attach to the membrane module and might play an important role in the contribution of membrane fouling.

In addition, the abundance variations of two types of functional microorganisms (AOB and NOB) involved in nitrification were analyzed, and five types were detected using high-throughput sequencing, including *Nitrosomonas* and *Nitrosococcus* involved in ammonia oxidation as well as *Nitrospira*, *Nitrobacter*, and *Nitrospina* involved in nitrite oxidation. Some studies have demonstrated that the AOB of different genera and different species of the same genus exhibit differences in physiology and adaptability to environmental factors, such as salinity and substrate content [62]. Among the AOB in activated sludge, *Nitrosomonas* usually has a higher abundance than *Nitrosococcus* and *Nitrosospira* [63,64]. *Nitrobacter* and *Nitrospira* are the two most common types of NOB in wastewater treatment. Under low NO_2_^−^-N concentrations, *Nitrospira* is often the dominant strain of NOB and has a higher abundance than *Nitrobacter* [65]. However, the distribution of AOB and NOB in the cake layer was very different from that in the sludge. Only *Nitrosococcus* and *Nitrospina*, but not the other three types of nitrifying microorganisms, were detected in the cake layer. The nitrification microbes in the cake layer also played a specific role in the removal of NH_4_^+^-N and NO_2_^−^-N.

**Figure 7:**
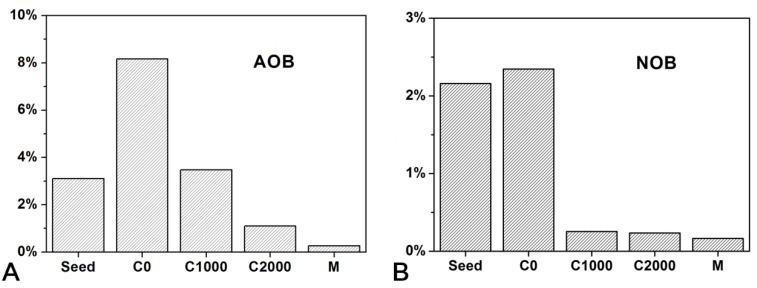
Variation of the relative abundance of (**A**) AOB and (**B**) NOB.

Figure 7A shows the relative abundances of AOB in the samples. The proportion of AOB in seed sludge was 3.11% and increased to 8.17% in the sludge sample with an influent COD of 0 mg/L. An environment with a low COD concentration was beneficial to the growth of AOB as autotrophs, leading to its enrichment in the C0 sample. As the influent COD content increased, the proliferation of heterotrophs inhibited AOB, resulting in a gradual reduction in the relative abundance of AOB in the C1000 and C2000 samples, accounting for only 3.48 and 1.10% of the total bacteria, respectively, which declined by 57.53 and 86.54% compared with the C0 sample, respectively. (The percentage of abundance difference was calculated by dividing the abundance difference between the C1000 or C2000 and C0 samples by the abundance in the C0 sample, which is the same below.) These findings were consistent with the results of Xia et al. [23]. The AOB exhibited the lowest abundance in the cake layer of the membrane module, accounting for only 0.26% of the bacteria. This finding might be attributed to the fact that the DO in the cake layer was reduced compared with that in the mixture, which was not conducive to the growth of aerobic bacteria, such as AOB. The abundance of NOB in each sample was similar to that of AOB (Figure 7B). Specifically, the abundance increased in the C0 sample, diminished significantly in the C1000 and C2000 samples, and reached the lowest in the cake layer. Similar to AOB, NOB are autotrophic bacteria that are also inhibited by the competition of heterotrophs in the process of COD promotion, causing a gradual reduction in the proportion of bacteria. The abundance of NOB in the C1000 and C2000 samples decreased by 89.32 and 90.17%, respectively, compared with the C0 sample.

## 4. Conclusions

The responses of the operational performance of an MBR with a high NH_4_^+^-N load and microbial community structure to increasing C/N ratios were studied. The results showed that the removal efficiencies of COD and NH_4_^+^-N in MBR were not affected by the variation in the influent C/N ratio, but the ammoxidation activity of sludge was gradually reduced. The stable removal of NH_4_^+^-N was ensured by the continuous increase in the sludge concentration in the reactor, which was four-fold increased at the end compared with that of the seed sludge. High influent COD stress promoted the production of humic acid SMP, which accelerated the membrane fouling rate. The average running time of the membrane module decreased to 7 days when the influent COD reached 2000 mg/L. Illumina-MiSeq sequencing showed that high influent COD stress increased the microbial diversity of sludge. The microbial composition of the filter cake layer was quite different from that of the sludge in the reactor, and microorganisms involved in nitrification were also found in the filter cake layer. The relative abundances of both the AOB and the NOB involved in nitrification declined gradually and were ultimately decreased by 86.54 and 90.17%, respectively, in the sample with an influent COD of 2000 mg/L compared with the sample with an influent COD of 0 mg/L.

Environmental factors have a significant impact on the varieties and distributions of nitrifiers, and the removal of NH_4_^+^-N under different external conditions is dominated by different kinds of nitrifiers. Looking forward to the future research directions, nitrifiers can be screened from different types of high NH_4_^+^-N wastewater biological treatment systems in order that functional bacterial agents for different types of wastewater can be developed and studied. Membrane fouling is still the main limiting factor of MBR. How to effectively control membrane fouling is the bottleneck of using MBR to treat high NH_4_^+^-N wastewater.

## Figures and Tables

**Table 1:** Operation conditions of the MBR system.

Stage	Time (Day)	COD (mg/L)	NH_4_^+^-N (mg/L)	C/N Ratio	ALR (gNH_4_^+^-N/L·Day)	HRT (h)	DO (mg/L)
I	1–25	0	1000	0	1.71	14	2–6
II	26–50	500	1000	0.5	1.71	14	2–6
III	51–75	1000	1000	1	1.71	14	2–6
IV	75–100	2000	1000	2	1.71	14	2–6

**Table 2:** Mean, standard deviation (SD), and variance analysis of effluent parameters under different C/N ratios. The abnormal data caused by the failure of the aeration system on the 16th and 76th days were excluded.

Effluent Parameters (mg/L)	Mean ± SD				*p*-Value
	C/N = 0	C/N = 0.5	C/N = 1	C/N = 2	
COD	10.39 ± 4.54	18.88 ± 7.94	28.53 ± 8.67	32.20 ± 8.56	0.000
NH_4_^+^-N	0.70 ± 0.21	0.52 ± 0.30	0.59 ± 0.47	0.68 ± 0.34	0.332
NO_2_^−^-N	2.69 ± 8.21	1.01 ± 1.95	5.25 ± 16.20	1.31± 3.07	0.394
NO_3_^−^-N	911.98 ± 34.32	911.08 ± 38.38	890.50 ± 52.34	919.84 ± 25.95	0.065

**Table 3:** Fluorescence spectral parameters of SMP in the effluent of the MBR.

COD (mg/L)	Peak A		Peak B	
	Ex/Em (nm) ^1^	Intensity ^2^	Ex/Em (nm)	Intensity
0	260/460	3197	345/432	1924
500	260/461	5880	350/437	3900
2000	260/464	6923	345/426	5211

^1^ Ex and Em represent excitation wavelength and emission wavelength, respectively. ^2^ Intensity represents the relative intensity of fluorescence, dimensionless.

## Data Availability

The data that support the findings of this study are available from the corresponding author (Z.W.) upon reasonable request.
